# ERA Registry Figure of the month Trends in kidney transplantation rate across countries

**DOI:** 10.1093/ckj/sfaf055

**Published:** 2025-03-22

**Authors:** Vianda S Stel, Alberto Ortiz, Anneke Kramer

**Affiliations:** ERA Registry, Department of Medical Informatics, Amsterdam UMC – Location University of Amsterdam, Amsterdam, the Netherlands; Amsterdam Public Health Research Institute, Quality of Care, Amsterdam, the Netherlands; Department of Nephrology and Hypertension, IIS-Fundacion Jimenez Diaz UAM, Madrid, Spain; Department of Medicine, Universidad Autonoma de Madrid, Madrid, Spain; ERA Registry, Department of Medical Informatics, Amsterdam UMC – Location University of Amsterdam, Amsterdam, the Netherlands; Amsterdam Public Health Research Institute, Quality of Care, Amsterdam, the Netherlands

**Figure 1:**
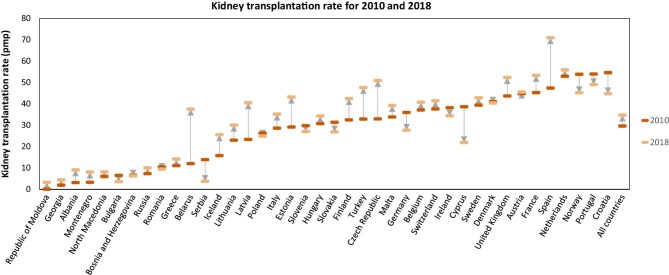
Kidney transplantation rate in 2010 and 2018 for 40 European countries, overall, and by country. **Source:** Boenink et al. NDT 2023. https://doi.org/10.1093/ndt/gfac333, Fig. 1A. The figure was slightly adapted from the original figure to show full country names. **Explanation:** For all countries combined the kidney transplant (KT) rate increased from 29.6 pmp in 2010 to 34.7 pmp in 2018, mainly due to an increase in the deceased donor KT rate (3.4 pmp) and to a lesser extent by an increase in the living donor KT rate (1.5 pmp). The KT rate varied widely across Europe. In sixteen countries (40%) the KT rate rose and in six countries (15%) the KT rate decreased during (a part of) the study period. Spain already had one of the highest KT rates in Europe in 2010, and due to a relatively high increase in the KT rate, it had the highest KT rate in 2018 (70.9 pmp).

